# 7-Hydroxycoumarin Attenuates Colistin-Induced Kidney Injury in Mice Through the Decreased Level of Histone Deacetylase 1 and the Activation of Nrf2 Signaling Pathway

**DOI:** 10.3389/fphar.2020.01146

**Published:** 2020-07-28

**Authors:** Jian Wang, Muhammad Ishfaq, Qianqian Fan, Chunli Chen, Jichang Li

**Affiliations:** ^1^College of Veterinary Medicine, Northeast Agricultural University, Harbin, China; ^2^Heilongjiang Key Laboratory for Animal Disease Control and Pharmaceutical Development, Northeast Agricultural University, Harbin, China

**Keywords:** colistin, Nrf2, HDAC1, 7-Hydroxycoumarin, renal injury

## Abstract

Colistin has been considered as the last line of defense against Gram-negative bacterial infections, however, the potential nephrotoxicity limited its clinical use. 7-Hydroxycoumarin (7-HC) possesses many beneﬁcial pharmacological activities. This study aimed to investigate the nephroprotective effects of 7-HC against colistin-induced kidney injury. *In vivo* experiments showed that 7-HC alleviated kidney injury induced by colistin, as indicated by lower levels of serum neutrophil gelatinase-associated lipocalin, blood urea nitrogen and creatinine levels. Both *in vivo* and *in vitro* results demonstrated that 7-HC alleviated oxidative stress and apoptosis induced by colistin, as shown by decreased malondialdehyde levels, decreased caspase-3 and caspase-9 activities, and increased superoxide dismutase and catalase activities. We also found that colistin significantly induced histone deacetylase (HDAC) 1 expression that deacetylated histone 3 at Lys27 acetylation (H3K27AC) of Nrf2 promoter region and hence inhibiting Nrf2 signaling. 7-HC treatment restored histone acetylation at the Nrf2 promoter region and hence promoted Nrf2 expression. These results suggested that 7-HC alleviates colistin-induced renal injury and this eﬀect was achieved by enhancement of renal antioxidant capacity with the decreased level of HDAC1 and the activation of Nrf2 signaling pathway.

## Introduction

In the past 20 years, there has been a pronounced increase in the emergence of multiple resistance of Gram-negative bacterial infections, especially multiresistant *Klebsiella pneumoniae*, *Acinetobacter baumannii* and *Pseudomonas aeruginosa* (Lachiewicz et al., 2017; Tissera et al., 2017). Due to the slow developmental process of new antibiotics, colistin has been considered as the last-line therapy for the treatment of multiresistant Gram-negative bacterial infections (Li et al., 2006). Dose-dependent nephrotoxicity is one of the main limiting factors that limit effective colistin therapy (Dai et al., 2015), and the clinical incidence of nephrotoxicity is 15%–60% (Kubin et al., 2012; Akajagbor et al., 2013; Ritesh and Arun, 2018). Therefore, it is necessary to develop effective nephroprotective agents for optimizing clinical use of colistin.

Understanding the mechanism of colistin-induced kidney injury is crucial for the development of nephroprotective agents. Colistin-induced kidney injury is characterized by increased generation of reactive oxygen species (ROS), which results in oxidative stress and apoptosis (Dai et al., 2015; Dai et al., 2017). Nuclear factor erythroid 2-related factor 2 (Nrf2) is an important transcription factor that regulates antioxidant genes, such as heme oxygenase-1 (HO-1), superoxide dismutase (SOD) and catalase (CAT) that alleviate oxidative stress (Zoja et al., 2014; Dai et al., 2015). It has been reported that the activation of Nrf2 signaling can relieve renal damage caused by colistin (Dai et al., 2015; Dai et al., 2017). In addition, the accumulated evidences have emphasized the importance of histone deacetylase (HDAC)–mediated epigenetic processes in the development of various renal diseases including drug-induced kidney injury (Wang et al., 2014; Ma et al., 2017; Yoshikazu et al., 2018). For example, sirtuin 7, a member of HDACs, knockout ameliorates cisplatin-induced acute kidney injury through regulating the nuclear expression of transcription factor NF-κB that resulted in decreased TNF-α expression (Yoshikazu et al., 2018). Overexpression of HDAC2 exacerbates cisplatin-induced renal tubular cells apoptosis through inhibiting the expression levels of BMP-7 epigenetically (Ma et al., 2017). Therefore, clarifying the role of HDAC in colistin-induced kidney injury and targeting HDAC may contribute to develop effective nephroprotective agents against colistin-induced kidney injury.

7-Hydroxycoumarin (7-HC) is a coumarin compound that is commonly found in Chinese herbs and vegetables (Wu et al., 2020). Previous studies reported the beneﬁcial pharmacological activities of 7-HC in different experimental models such as protective effects against methotrexate- and cisplatin-induced renal injury (Hassanein et al., 2018; Wu et al., 2020), liver damage caused by carbon tetrachloride (Mahmoud et al., 2019), myocardial infarction induced by isoproterenol (Jagadeesh et al., 2016) and cerebral ischemia-reperfusion-induced injury (Wang et al., 2015). Therefore, 7-HC has great potential to develop as functional food or nutritional supplement (Wu et al., 2020). Moreover, 7-HC may be a potential adjuvant therapeutic drug that could enhance the antibacterial effects of antibiotics. Previous studies showed that 7-HC could affect the motility and quorum-sensing (QS) activity of *Escherichia coli* and *Staphylococcus aureus* (these bacteria are commonly found in hospital-acquired infections), which indicated that 7-HC plays an important role in the interference of cell-cell interactions and in biofilm formation (Lee et al., 2014; Monte et al., 2014).

In the present study, we demonstrate that 7-HC is an effective nephroprotective agent against colistin-induced kidney injury and the protective eﬀect was achieved through the decreased level of HDAC1 and the activation of Nrf2 signaling pathway.

## Materials and Methods

### Reagents and Antibodies

Colistin (sulfate) was obtained from Zhejiang Shenghua Biology Co., Ltd (20400 U/mg, Zhengjiang, China). 7-HC (purity 99%) was purchased from National Institutes for Food and Drug Control (Beijing, China). Primary antibodies against Lamin B (catalog number: ab169306), Nrf2 (catalog number: ab31163), HO-1 (catalog number: ab13248) and histone 3 at Lys27 acetylation (H3K27AC) (catalog number: ab4729) were purchased from Abcam (Cambridge, MA, UK). Primary antibodies against HDAC1 (catalog number: 34589S) was purchased from Cell Signaling Technology (Danvers, MA, USA). Anti-β-actin (catalog number: sc-8432) and horseradish peroxidase (HRP)-labeled goat anti-rabbit IgG (catalog number: sc-2030) were purchased from Santa Cruz Biotechnology (Santa Cruz, CA, USA).

### Animal Experiments

The animal experiments were approved by the Institutional Animal Care and Use Committee of Northeast Agricultural University. Adult Kunming mice (female, 6 to 8 weeks, 18 to 22 g) were obtained from Experimental Animal Centre of Harbin Veterinary Research Institute of Chinese Academy of Agricultural Sciences (Harbin, China). The animal laboratory was maintained at approximately 22°C and 50% relative humidity with a 12 h light-dark cycle. After 1 week of acclimation, mice were randomly divided into four groups (n=6 in each group): saline solution (control group); 7-HC administered at 50 mg/kg of body weight/day (7-HC group); colistin at 15 mg/kg/day (colistin group); colistin at 15 mg/kg/day plus 7-HC at 50 mg/kg/day (colistin + 7-HC group). For colistin treatment, mice were intraperitoneally injected colistin (colistin sulfate in sterile saline) *via* a 3-min infusion as described previously (Dai et al., 2015). In the colistin + 7-HC group, mice were intraperitoneally injected 7-HC (7-HC in 5% DMSO) at 2 h before colistin treatment. Mice in control or 7-HC group were given an equal volume of saline solution or 5% DMSO in saline, respectively. All mice were treated for 7 consecutive days and euthanized by cervical dislocation at 12 h after the last dose. The kidney tissues were immediately collected and frozen by liquid nitrogen, and then stored at −80°C for further analysis. Serum was collected from blood samples at room temperature for 45 min to allow coagulation and subsequently centrifuged at 3,500 × g for 15 min. Supernatant was harvested and stored at −80°C for further analysis.

### Histopathological Examination

Histopathological examination was performed as described previously (Dai et al., 2015). Briefly, the left kidney tissue samples were randomly collected from four mice of each group and fixed with 10% neutral buffered formalin at least 12 h. The formalin-ﬁxated tissues were dehydrated in a series of graded alcohols, xylene transparency and embedded in paraffin wax. Paraffin sections (4 μm) were stained with hematoxylin and eosin (HE), and then examined under an optical microscope. A semiquantitative score (SQS) was used to grade the lesion severity for each kidney sample. Scores of 0, + 1, +2, +3, +4, and +5 corresponded to no change, mild change, mild to moderate change, moderate change, moderate to severe change, and severe change, respectively.

### Measurement of Kidney Function

Blood urea nitrogen (BUN) and creatinine (CRE) levels were measured using standard detection kits according to the manufacturer’s instructions (Jiancheng Bioengineering Institute, Nanjing, China). Serum neutrophil gelatinase-associated lipocalin (NGAL) were measured using Mouse NGAL Quantikine ELISA Kit (R&D Systems China Co. Ltd., Changning, China) according to the manufacturer’s instruction.

### Measurement of Oxidative Stress Markers

Samples from each group were collected and lysed using the cell lysis buffer provided by the manufacturer. Centrifugation was performed at 3,000 × g (4°C) for 15 min to get the supernatant. The concentrations of malondialdehyde (MDA), CAT, and SOD activities were determined by using commercial kits (Jiancheng Bioengineering Institute, Nanjing, China). Protein concentrations in the supernatant were adjusted using the bicinchoninic acid (BCA) Kit (Beyotime, Haimen, China).

### Measurement of Caspase-3/Caspase-9 Activities

Caspase-3 and caspase-9 activities were measured by using commercial detection kits (Jiancheng Bioengineering Institute, Nanjing, China). Samples from each group were collected and lysed using the cell lysis buffer provided by the manufacturer. Centrifugation was performed at 12,000 × g (4°C) for 15 min to get the supernatant. The supernatants were used to determine the activities of caspase-3 and caspase-9. Protein concentrations in the supernatant were adjusted by using the BCA Kit.

### Western Blotting

Western blotting was carried out as previously described (Wang et al., 2019). Nuclear proteins were extracted using a Nuclear Protein Extraction Kit (Solarbio, Beijing, China) and total protein were prepared in radio-immunoprecipitation assay (RIPA) lysis buffer containing 0.5 mM PMSF (Beyotime, Haimen, China), and centrifuged at 12,000 × g for 15 min at 4°C. Equivalent amounts of protein were separated in a 10-12% gel by SDS-polyacrylamide gel electrophoresis (SDS-PAGE) and then transferred onto nitrocellulose (NC) membranes. The NC membranes were blocked with 5% skim milk dissolved in TBST (Solarbio, Beijing, China) at 4°C for 1 h and incubated overnight with primary antibodies. The secondary antibody was incubated at room temperature for 1 h and the blots were detected by BeyoECL Plus (Beyotime, Haimen, China). The results were assessed by an image capture and analysis system (GeneGnome, Syngene, UK).

### RNA Sequencing

Kidney tissue samples were randomly collected from three mice of each group. RNA preparation, library construction and sequencing were performed on the BGISEQ-500 platform at the Beijing Genomics Institute (BGI, Shenzhen, China). The sequencing data was filtered with SOAPnuke (v1.5.2) and the clean reads were mapped to the reference genome using HISAT2 (v2.0.4). Bowtie2 (v2.2.5) was applied to align the clean reads to the reference coding gene set, then the expression level of gene was calculated by RSEM (v1.2.12). The Venn map was drawn by Venn Diagram (v1.6.20) according to the gene expression in different samples. The protein-protein interaction (PPI) network was predicted using Search Tool for the Retrieval of Interacting Genes (STRING; http://string-db.org) (version 10.0) online database. Essentially, differential expression analysis was performed by using the DESeq2 (v1.4.5) with fold change ≥ 2 and q value ≤ 0.001. RNA sequencing (RNA-seq) data were deposited in the Gene Expression Omnibus (Accession no. GSE150518).

### Quantitative Real-Time PCR Analysis

The procedure was carried out as described previously (Wang et al., 2019). Briefly, total RNA was extracted using Trizol reagent (Life Technologies, Grand Island, NY, USA) according to the manufacturer’s instructions. Total RNA was qualified and quantified using a Nano Drop and Agilent 2100 bioanalyzer (Thermo Fisher Scientific, MA, USA). One migcrogram of total RNA (the RNA integrity number ≥ 8 and concentration ≥ 50 ng/μl) were reverse transcribed using the Transcriptor First Strand cDNA Synthesis Kit (Transgen, Beijing, China). The gene expression levels were detected using real-time PCR with a SYBR premix Ex Taq kit (Transgen, Beijing, China) on an Applied Biosystems 7500 real-time PCR system thermocycler. Each sample was analyzed in triplicates and the mRNA expression of the target genes was analyzed by 2^−△△Ct^ method, following normalization with β-actin gene. Primer sequences of HDAC1 and β-actin genes are shown as follows: HDAC1, 5’-TGAAGCCTCACCGAATCCG-3’ and 5’-GGGCGAATAGAACGCAGGA-3’ (Deng et al., 2020); β-actin, 5’-GTTGGAGCAAACATCCCCCA-3’ and 5’-ACGCGACCATCCTCCTC TTA-3’ (Yu et al., 2018).

### Cell Culture and Treatments

Mouse renal tubular epithelial cells (mRTECs) were obtained from the Cell Bank of the Type Culture Collection, Shanghai Institute of Cell Biology, Chinese Academy of Sciences. The cells were grown in DMEM supplemented with 10% (vol/vol) FBS at 37°C in a humidified atmosphere containing 5% CO_2_. Experimental groups including control group (cells incubated serum-free DMEM); 7-HC group (cells pretreated with 20 μM 7-HC for 2 h, then incubated with serum-free DMEM); colistin group (cells incubated 200 µM colistin for 24 h); colistin + 7-HC group (cells pretreated with 20 μM 7-HC for 2 h, then coincubated with 200 µM colistin for 24 h); control + siHDAC1 group (cells pretransfected with siRNA_HDAC1, then incubated with serum-free DMEM); colistin + siHDAC1 group (cells pretransfected with siRNA_HDAC1, then incubated with 200 µM colistin for 24 h). After colistin treatment, the cells were collected for further analysis and all experiments were conducted at least three times.

### Knockdown of HDAC1 in mRTECs

The cells were preftransfected with siRNA_HDAC1 using Lipofectamine 2000TM reagent (Invitrogen, Carlsbad, CA, USA). Negative control siRNA was used as a control. siRNAs targeting HDAC1 were purchased from GenePharma Co. Ltd (Shanghai, China). The siRNA sequences used in experiments are as follows: siRNA_1_HDAC1 (sense: 5’-GCCGGUCAUGCUCAAAGUATT-3’ and antisense: 5’-UACUUUGGACAUGACCGGCTT-3’); siRNA_2_HDAC1 (sense: 5’-CCACCGC AAUUCAUUUAGUTT-3’ and antisense: 5’-ACUAAAUGAAUUGCGGUGGTT-3’). Negative control siRNA (sense: 5’-UUCUCCGAACGUGUCACGUTT-3’ and antisense: 5’-ACGUGACACGUUCGGAGAATT-3’). Transfection efficiency was evaluated by Western blot.

### MTT Assay

Cells were seeded in 96-well plates. After culture for 12 h, cells were pretreated with 7-HC (0–40 µM), and colistin (0 or 200 µM) for 24 h. The medium was removed and replaced with serum-free medium containing 5 mg/ml MTT and the cells were incubated for 4 h at 37°C. Finally, the medium was discarded and incubated with DMSO for 20 min at room temperature. The absorbance was read at 570 nm in a microplate reader (Molecular Devices, Sunnyvale, CA, USA).

### ROS Measurement

ROS content of mRTECs were detected using the ROS-specific fluorescent dye 2’, 7’-Dichlorofluorescensin diacetate (DCFH-DA, Beyotime, Haimen, China) according to our previous study (Wang et al., 2019). Briefly, cells were collected from each group and washed three times with cold PBS, then DCFH-DA was added into the medium for a further 45 min. Fluorescence was imaged using a fluorescent microscope at excitation wavelength 488 nm, emission wavelength 530 nm (Olympus, Japan).

### HDAC Activity Assay

The procedure was carried out as described previously (Andrade-Oliveira et al., 2015). Each group samples were collected and total protein extracts were prepared in radio-immunoprecipitation assay (RIPA) lysis buffer containing 0.5 mM PMSF, then centrifuged at 12,000 × g for 15 min at 4°C. Equivalent amounts of protein from each group were used to measure HDAC activity in an HDAC activity colorimetric assay kit according to the manufacturer’s (Biovision) instructions.

### Chromatin Immunoprecipitation Assay

Cells were cross-linked by 1% formaldehyde and the procedure of chromatin immunoprecipitation (ChIP) was carried out according to the manufacturers’ instructions by using Magna ChIPHiSens Kit (Millipore, Darmstadt, Germany) as described previously (Liu et al., 2017). Chromatin DNA samples were immunoprecipitated with antibodies against mouse IgG, H3K27AC or HDAC1. PCR amplification was performed in 50 μl volumes for 30–35 cycles to determine the appropriate conditions for the PCR products of Nrf2 promoter region. Primer sequences as follows: Nrf2-promoter F: 5’-TCCTCTGGGGGCATGGTAAT-3’; Nrf2-promoter R: 5’-GTTCTTGCGTGCT CCAGATG-3’.

### Statistical Analysis

Data were obtained from at least three independent experiments and are presented as means ± SD. One-way analysis of variance (ANOVA) was used for analysis of data, followed by the LSD *post hoc* test using SPSS v. 21.0 (SPSS Inc., Chicago, IL, USA). A value of *P* < 0.05 was regarded as statistically significant.

## Results

### 7-HC Ameliorates Colistin-Induced Kidney Injury in Mice

**Figure 1:**
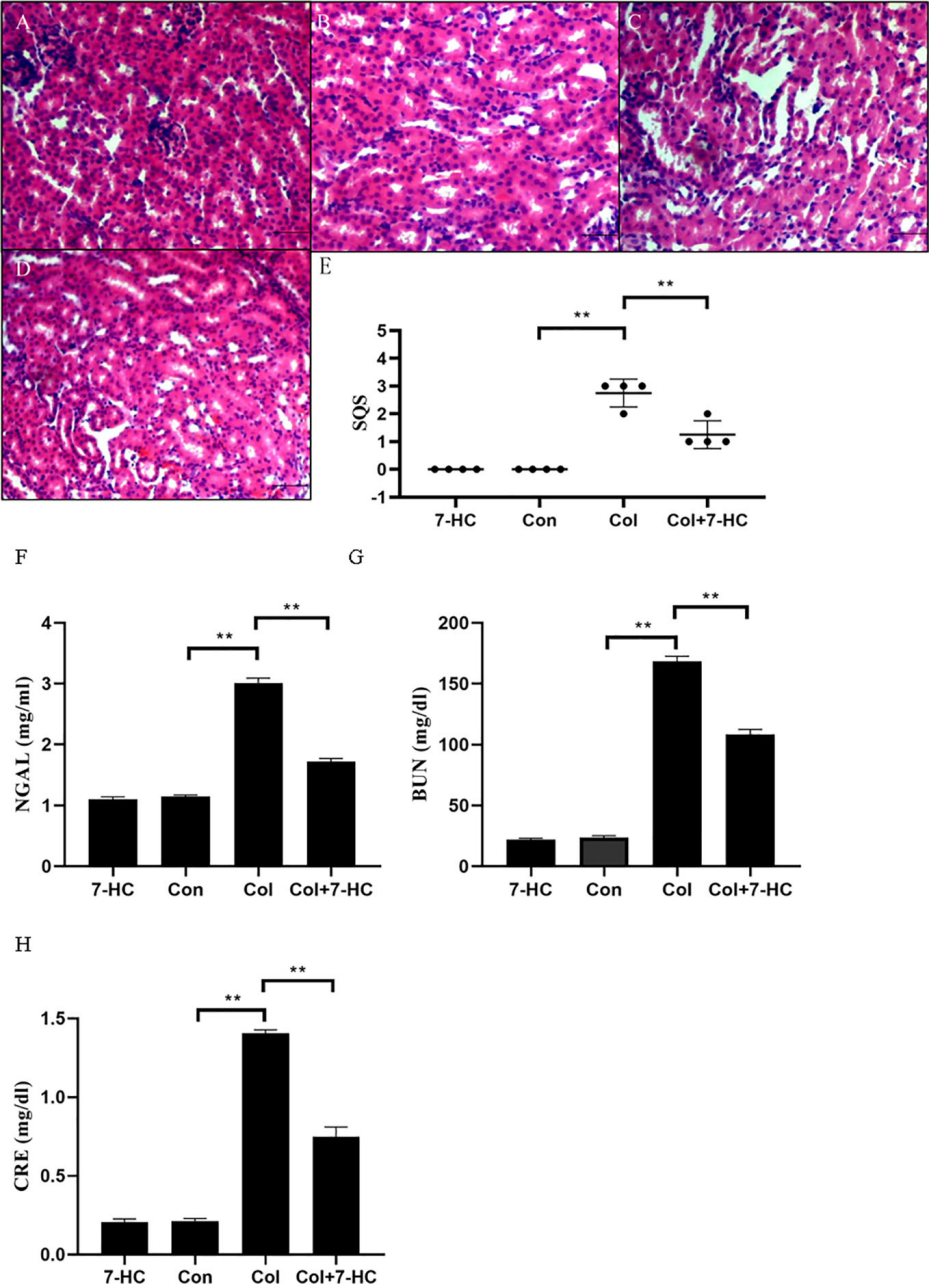
7-Hydroxycoumarin (7-HC) alleviates kidney injury and the loss in renal function in colistin-induced kidney injury mice. **(A)** Hematoxylin and eosin (HE) staining of kidneys in the 7-HC group, no marked injury. **(B)** HE staining of kidneys in the control group, no marked injury. **(C)** HE staining of kidneys in the col group, extensive damage seen as renal tubule amplification, epithelial cells detachment, and necrosis. **(D)** HE staining of kidneys in the col + 7-HC group, mild tubular damage with necrosis of epithelial cells and cast formations. **(E)** Semiquantitative score (SQS) values that represent histopathological changes (n = 4). **(F–H)** Levels of serum neutrophil gelatinase-associated lipocalin (NGAL), Blood urea nitrogen (BUN) and creatinine (CRE), respectively (n = 6). Values are the mean ± SD that are significantly different indicated by asterisks as follows: ^**^P < 0.01. Bars, 100 μm. Con, control; Col, colistin.

In the control group and 7-HC group, no marked histopathological changes of kidney tissues were observed (**Figures 1A, B**). However, kidney tissues in the colistin group exhibited extensive damage such as renal tubule amplification, epithelial cells detachment and necrosis (**Figure 1C**); the SQS increased to 2.75 ± 0.50 (**Figure 1E**, *P* < 0.01). In the colistin + 7-HC group, these histopathological changes were markedly attenuated (**Figure 1D**); the SQS decreased to 1.25 ± 0.50 (**Figure 1E**, *P* < 0.01). Serum NGAL, BUN, and CRE levels were increased in response to impairment in renal function. Compared to the control group, serum NGAL, BUN, and CRE levels did not significantly changed in the 7-HC group but markedly increased in the colistin group (**Figures 1F–H**, all *P* < 0.01). In the colistin + 7-HC group, pretreatment with 7-HC effectively attenuated the increase in serum NGAL, BUN and CRE levels (**Figures 1F–H**, all *P* < 0.01).

### 7-HC Ameliorates Colistin-Induced Oxidative Stress and Apoptosis in Kidney Tissue

**Figure 2:**
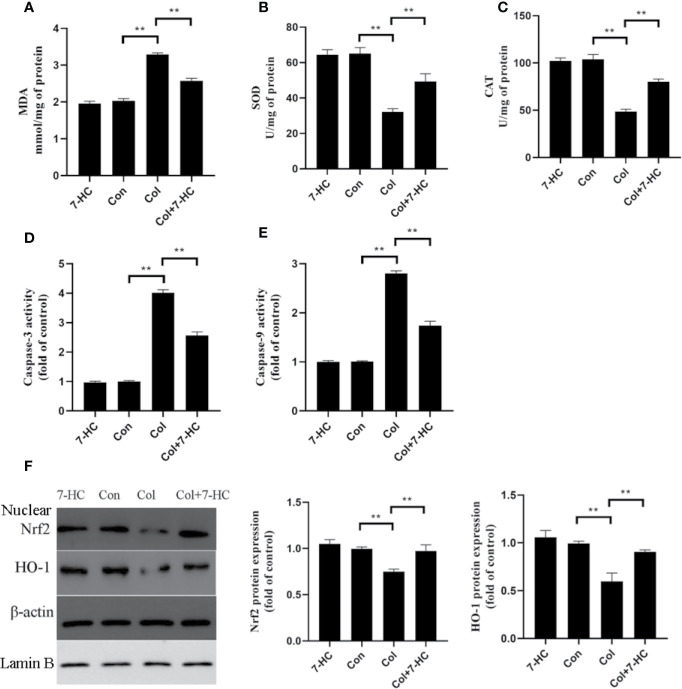
7-Hydroxycoumarin (7-HC) significantly alleviates oxidative stress and apoptosis in colistin-induced kidney injury mice. **(A–C)** The levels of oxidative stress markers in the kidney tissues of mice in each group (n = 6). **(D, E)** Activities of caspase-3 and caspase-9 in the kidney tissues of mice in each group (n = 6). **(F)** Protein expression of Nrf2 and HO-1 in the kidney tissues of mice in each group (n = 3). Values are the mean ± SD that are significantly different indicated by asterisks as follows: ^**^P < 0.01. Con, control; Col, colistin.

Compared to control group, colistin treatment markedly increased the level of MDA and significantly reduced the activities of SOD and CAT, respectively (**Figures 2A–C**, all *P* < 0.01). Compared to colistin group, pretreatment with 7-HC significantly attenuated all of these colistin-induced biomarkers of oxidative stress (**Figures 2A–C**, all *P* < 0.01). These oxidative stress biomarkers did not significantly change between the 7-HC and control groups (**Figures 2A–C**). Simultaneously, the caspase-3 and caspase-9 activities in col group significantly increased compared to control group, whereas 7-HC treatment significantly suppressed the increased activities of caspase-3 and caspase-9 compared to col group (**Figures 2D, E**, all *P* < 0.01). Furthermore, colistin treatment signiﬁcantly decreased protein expression of Nrf2 and HO-1 compared to control group, while 7-HC treatment significantly promoted the activation of Nrf2 and HO-1 signaling compared to colistin group (**Figure 2F**, all *P* < 0.01).

### Hub Genes Selection by RNA-Seq

**Figure 3:**
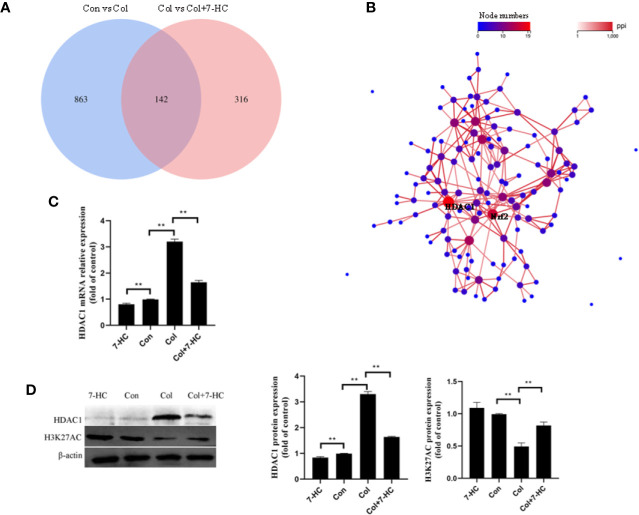
Hub genes selection and identification. **(A)** Venn diagram, two comparison groups showed an overlap of 142 different expressed genes. **(B)** The protein-protein interaction (PPI) network of overlapped different expressed genes, HDAC1 and Nrf2 were considered the key genes due to the most nodes. **(C)** HDAC1 mRNA expression levels in kidney tissues by quantitative real-time PCR (qRT-PCR) (n = 6). **(D)** Protein expression of HDAC1 and H3K27AC in kidney tissues (n = 3). Values are the mean ± SD that are significantly different indicated by asterisks as follows: ^**^P < 0.01. Con, control; Col, colistin.

To further explore the mechanism of 7-HC protection against colistin-induced kidney injury, RNA-seq were performed. 1005 and 458 different expression genes were identified between control vs colistin and colistin vs colistin + 7-HC, respectively (**Figure 3A**). The overlap between the two comparison groups contained 142 genes as shown in the Venn diagram (**Figure 3A**). PPI network of different expression genes may provide insights into the mechanisms of generation or development of diseases (Li et al., 2017). HDAC1 and Nrf2 were identified as hub genes because they are at the heart of the network of the 142 differentially expressed genes (**Figure 3B**). Consistent with the result of RNA-seq, colistin-treatment significantly increased the mRNA and protein expression of HDAC1 compared to the control group, while 7-HC treatment significantly decreased the mRNA and protein expression of HDAC1 compared to the con or col group, respectively (**Figures 3C, D**, all *P* < 0.01). Changes in HDAC1 may affect acetylation modification. H3K27AC is a specific acetylation modification of histone 3 which could promote gene expression, such as Nrf2 (Yang et al., 2018). It has also been noted that the expression of H3K27AC was significantly reduced in the colistin group compared with the control group (**Figure 3D**, *P* < 0.01). Compared with the colistin group, the expression of H3K27AC was significantly increased in colistin + 7-HC group (**Figure 3D**, *P* < 0.01). Compared with the control group, no marked changes of H3K27AC were observed in 7-HC group (**Figure 3D**).

### 7-HC Ameliorates Colistin-Induced Cellular Damage, Oxidative Stress, and Apoptosis in mRTECs

**Figure 4:**
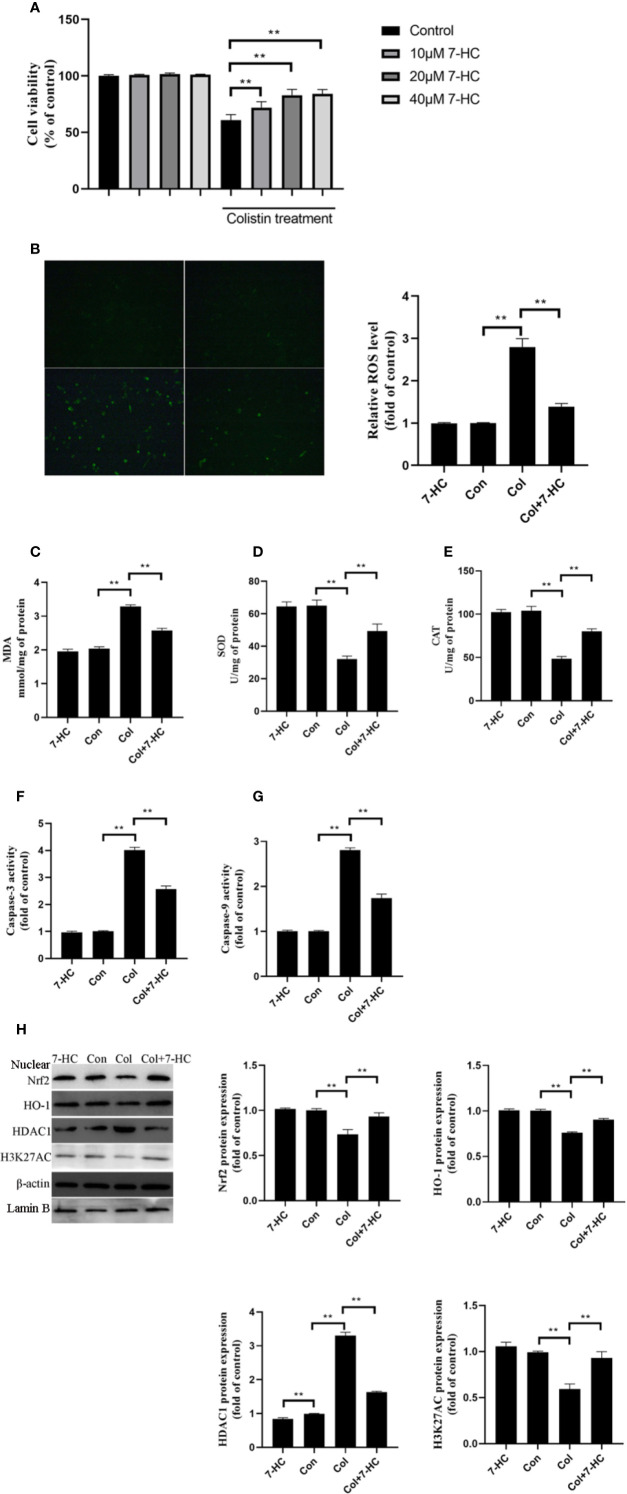
7-Hydroxycoumarin (7-HC) suppresses colistin-induced cellular damage, oxidative stress and apoptosis in mouse renal tubular epithelial cells (mRTECs). **(A)** Impact of 7-HC on colistin-induced cytotoxicity in mRTECs by MTT assay. **(B)** Cellular reactive oxygen species (ROS) levels of mRTECs in each group. **(C–E)** The levels of oxidative stress markers of mRTECs in each group. **(F, G)** Activities of caspase-3 and caspase-9 of mRTECs in each group. **(H)** Protein expression of Nrf2, HO-1, HDAC1, and H3K27AC in mRTECs. Values were represented by the mean ± SD from at least three independent experiments. Values that are significantly different indicated by asterisks as follows: ^**^P < 0.01. Con, control; Col, colistin.

To further verify the protective effect of 7-HC and clarify the underlying mechanism, cell experiments were carried out. 7-HC treatment per se had no impact on cell viability (**Figure 4A**). Treatment of mRTECs with 200 μM colistin for 24 h induced almost 40% decrease in cell viability (**Figure 4A**, *P* < 0.01). 10, 20, and 40 μM 7-HC treatment attenuates colistin-induced cytotoxicity (**Figure 4A**, *P* < 0.01). 20 and 40 μM 7-HC showed similar protective effects (**Figure 4A**), therefore, 20 μM 7-HC were used in subsequent experiments. Compared to control group, the colistin group displayed significantly increased ROS and MDA levels (**Figures 4B, C**, all *P* < 0.01), and significantly decreased SOD and CAT activities (**Figures 4D, E**, all *P* < 0.01). Compared to col group, colistin + 7-HC group showed significantly decreased ROS and MDA levels (**Figures 4B, C**, all *P* < 0.01), significantly increased SOD and CAT activities (**Figures 4D, E**, all *P* < 0.01). In addition, colistin treatment significantly increased caspase-3 and caspase-9 activities, while 7-HC treatment significantly suppressed the increased activities of caspase-3 and caspase-9 compared to colistin group (**Figures 4F, G**, all *P* < 0.01). Furthermore, colistin treatment signiﬁcantly increased expression of HDAC1, decreased protein expression of Nrf2, HO-1 and H3K27AC compared to control group, while col + 7-HC group showed significantly increased protein expression of the Nrf2, HO-1 and H3K27AC compared to colistin group (**Figure 4H**, all *P* < 0.01). 7-HC treatment significantly decreased the protein expression of HDAC1 compared to the control or colistin group, respectively (**Figure 4H**, all *P* < 0.01).

### Gene Silencing of HDAC1 Ameliorates Colistin-Induced Oxidative Stress and Apoptosis in mRTECs

**Figure 5:**
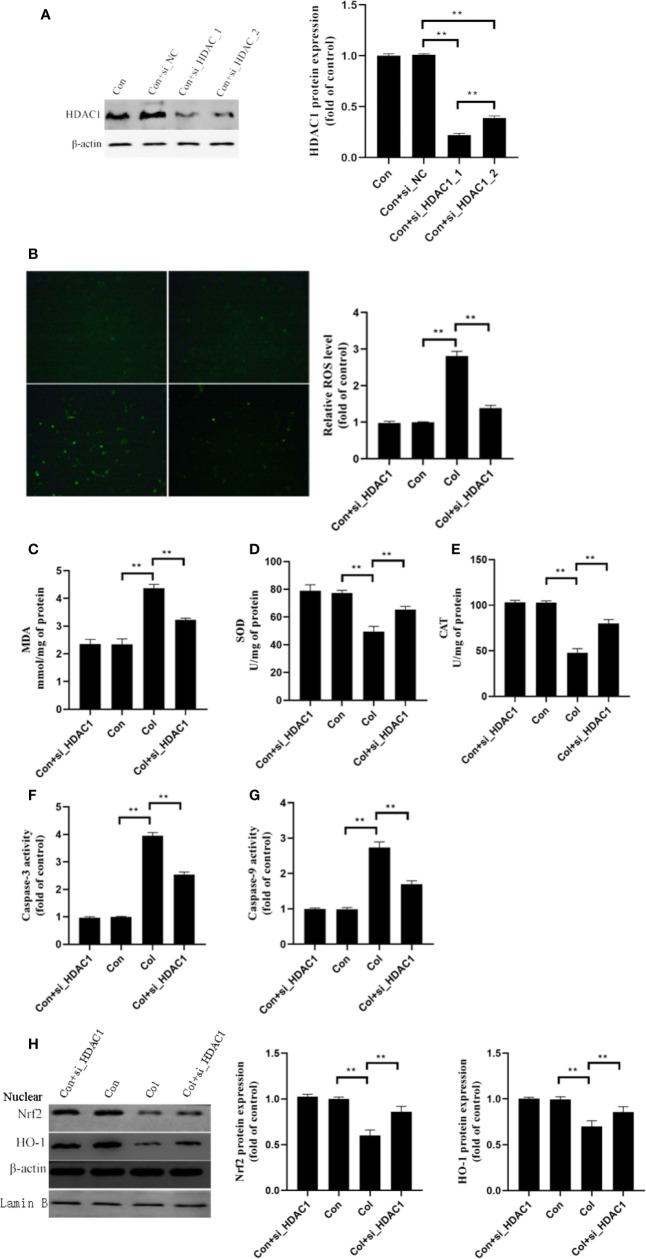
Gene silencing of HDAC1 ameliorates colistin-induced oxidative stress and apoptosis in mRTECs. **(A)** HDAC1 protein expression levels after HDAC1-siRNA transfection. **(B)** Cellular reactive oxygen species (ROS) levels of mRTECs in each group. **(C–E)** The levels of oxidative stress markers of mouse renal tubular epithelial cells (mRTECs) in each group. **(F, G)** Activities of caspase-3 and caspase-9 of mRTECs in each group. **(H)** Protein expression of Nrf2 and HO-1in mRTECs. Values were represented by the mean ± SD from at least three independent experiments. Values that are significantly different indicated by asterisks as follows: ^**^P < 0.01. Con, control; Col, colistin.

Based on the above results, we hypothesized that HDAC1 may be involved in the protective effect of 7-HC against colistin-induced renal injury. We further silenced the expression of HDAC1 by two siRNA in mRTECs (**Figure 5A**, *P* < 0.01). The control + siRNA_1_HDAC1 showed better performance in silencing of HDAC1, which was used in subsequent experiments. Compared to colistin group, gene silencing of HDAC1 significantly decreased ROS and MDA levels (**Figures 5B, C**, all *P* < 0.01), significantly increased SOD and CAT activities (**Figures 5D, E**, all *P* < 0.01). In addition, colistin treatment significantly increased caspase-3 and caspase-9 activities, while gene silencing of HDAC1 significantly suppressed the increased activities of caspase-3 and caspase-9 compared to colistin group (**Figures 5F, G**, all *P* < 0.01). Furthermore, gene silencing of HDAC1 did not affect the protein expression of Nrf2 and HO-1 compared to control group, while significantly increased protein expression of the Nrf2 and HO-1 compared to colistin group (**Figure 5H**, all *P* < 0.01).

### HDAC1 Inhibits the Expression of Nrf2 by Deacetylating H3K27AC

**Figure 6:**
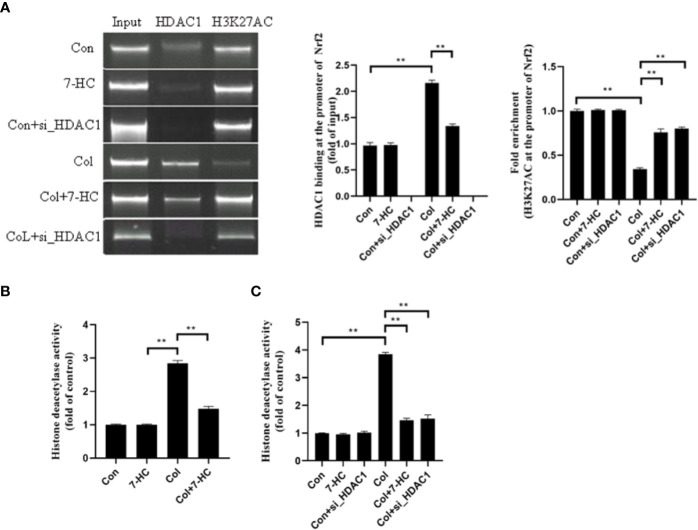
HDAC1 bound to the promoter region of Nrf2 and deacetylated H3K27AC thereby inhibiting Nrf2 expression. **(A)** Chromatin immunoprecipitation (ChIP) analysis was used to detect HDAC1 binding and H3K27AC at the promoter region of Nrf2 in mRTECs. The results are shown relative to background levels of IgG antibody (n = 3). **(B)** Measurement of HDAC activity of mice in each group (n = 6). **(C)** Measurement of HDAC activity of mRTECs in each group. Values are the mean ± SD that are significantly different indicated by asterisks as follows: ^**^P < 0.01. Con, control; Col, colistin.

Is the Nrf2 signaling mediated by HDAC1 related to the change of acetylation level at H3K27AC? To clarify this, we detected whether Nrf2 promoter region binds to HDAC1 by CHIP. We found that HDAC1binds to Nrf2 promoter region in mRTECs and this binding increased significantly after colistin treatment. 7-HC treatment could reduce the expression of HDAC1, thereby reducing the binding between HDAC1 and Nrf2 promoter region (**Figure 6A**, all *P* < 0.01). ChIP results also showed that the H3K27AC was bound to the Nrf2 promoter region. It was noted that the acetylation level of H3K27AC in the Nrf2 promoter region of mRTECs significantly decreased after colistin treatment. Colistin + 7-HC or colistin + siHDAC1 group showed markedly increased acetylation levels of H3K27AC in the Nrf2 promoter region compared to col group (**Figure 6A**, all *P* < 0.01). These results suggesting that the protective effect of 7-HC against colistin-induced renal injury involved the inhibition of HDAC1 and hence the activation of Nrf2 signaling. Furthermore, we also noticed that though 7-HC treatment or gene silencing of HDAC1 down-regulated HDAC1 expression that did not affect the protein expression of H3K27AC and Nrf2 (**Figures 2**–**6**). Therefore, we further examined the total HDAC activity. Compared to control group, colistin treatment significantly increased HDAC activity both in mice and mRTECs (**Figures 6B, C**, all *P* < 0.01). Colistin + 7-HC or colistin + siHDAC1 group displayed significantly down-regulated HDAC activity in mice or mRTECs, compared to the colistin group (**Figures 6B, C**, all *P* < 0.01). These results indicated that colistin-induced increased HDAC1 expression contributed to the increased HDAC activity. However, no markedly changed HDAC activities were observed in 7-HC or control + siHDAC1 group compared to control group (**Figures 6B, C**, all *P* < 0.01).

## Discussion

Clinically, one of the major side-effects of colistin is its potential nephrotoxicity risk (Ritesh and Arun, 2018). Therefore, the development of nephroprotective agents to reduce this unwanted side-effect is urgent until more effective therapeutic options for multidrug-resistant Gram-negative bacterial infections can be found. 7-HC, a member of coumarin family, commonly found in many plants such as Eucommiae Cortex and garden angelica (Ouyang et al., 2019; Wu et al., 2020). Recently, 7-HC has gained an increasing focus because of its extensive pharmacological properties such as anti-oxidant and anti-inflammation activities. In addition, previous studies have shown that 7-HC could alleviate hepatic injury and reduce the infarct size of the myocardium *via* attenuating oxidative stress (Govindan et al., 2016; Mahmoud et al., 2019). 7-HC supplementation was also shown to alleviate cisplatin-induced nephrotoxicity in mice (Wu et al., 2020). These studies suggested that 7-HC might be a potential molecule that can be used to reduce colistin-induced kidney injury. In the present study, we first evaluated the protective effects of 7-HC at different doses (10-100 mg/kg) against colistin-induced nephrotoxicity, and found that 7-HC suppressed the increase of serum NGAL, BUN and CRE in a dose-dependent manner (**Supplementary Figures 1A–C**). The 75 and 100 mg/kg of 7-HC groups showed significantly decreased serum NGAL, BUN, and CRE than (*P* < 0.01) the colistin group, but showed no signiﬁcant diﬀerence compared to the 50 mg/kg of 7-HC group. Based on the above results, 50 mg/kg of 7-HC was chosen for the following experiments. Here, we provide demonstrable proof that 50 mg/kg of 7-HC possess excellent nephroprotective effects against colistin by reducing ROS production and inhibiting oxidative stress.

Previous studies have shown a strong association of colistin-induced kidney injury to oxidative stress due to significantly increased ROS production in response to colistin exposure (Dai et al., 2015; Dai et al., 2017). In line with previous studies, we confirmed that colistin-induced kidney injury is relevant to markedly elevated ROS and MDA levels and concomitant reduced activities of antioxidant enzymes SOD and CAT (Dai et al., 2015; Wang et al., 2019). In the present study, 7-HC treatment effectively reduced the ROS contents and alleviated oxidative stress. The protective effects were also determined by pathological examination of kidney tissues of mice. Colistin could activate mitochondrial apoptosis pathway that is a key event in the nephrotoxicity of colistin (Dai et al., 2014). Caspase-3 and caspase-9 are crucial indicators in mitochondrial apoptosis pathway (Dai et al., 2015). Previous studies indicated that 7-HC could ameliorate myocardial injury following ischemia−reperfusion in the rat *via* inhibiting the mitochondrial apoptosis pathway (Luo et al., 2018). Our present study confirmed that 7-HC treatment significantly reduced the activities of caspase-3 and caspase-9, which may due to 7-HC treatment that minimized ROS production. The Nrf2 signaling is crucial in alleviating xenobiotic insults and many animal- and cell-based studies. Moreover, it has been confirmed that Nrf2 signaling inactivation was involved in drug-induced nephrotoxicity (Dai et al., 2015, Deng et al., 2010; Shelton et al., 2013; Li et al., 2019; Michel and Menze, 2019; Wang et al., 2019). In the present study, our results indicated that the activation of the Nrf2 signaling contributed to the ability of 7-HC to alleviate colistin-induced kidney injury. In addition, the expression of Kelch-like ECH-associated protein 1 (Keap1), the negative regulator of Nrf2, was not changed by 7-HC treatment both in control and colistin conditions (**Supplementary Figure 2**), which means that 7-HC could activate Nrf2 signaling *via* Keap1-independent mechanism.

Histone acetylation modification, regulated by HDAC, is an important mechanism for gene transcription regulation (Wang et al., 2014; Jing et al., 2017). Increasing evidences have emphasized the importance of HDAC-regulated epigenetic modification in the development of kidney injury (Wang et al., 2014; Ma et al., 2017; Yoshikazu et al., 2018). When exposed to ischemia/reperfusion injury, histone acetylation modification of kidneys was reduced, while β-hydroxybutyrate attenuates kidney injury by restoring histone acetylation at the FOXO3 promoter (Takaya et al., 2019). In addition, targeting histone H4 acetylation *via* phosphoinositide 3-kinase- and p70s6-kinase-dependent pathways could inhibit iNOS production and alleviated injury in glomerular mesangial cells (Yu and Kone, 2006). Therefore, inhibitors of HDAC are considered as promising nephroprotective agents. Previous studies that showed that cisplatin up-regulated the expression of HDAC1 in the kidney and HDAC1 specific inhibitor MS-275 reduced cisplatin-induced kidney injury by up-regulating AMWAP (an anti-inflammatory protein) protein expression (Ranganathan et al., 2016). In addition, treatment with romidepsin, a selective inhibitor of HDAC1, significantly attenuated lipopolysaccharide-induced acute kidney injury (Cheng et al., 2020). Similarly, in the present study, we found that HDAC1 is a key gene in colistin-induced kidney injury by RNA-seq. H3K27AC, a transcriptional activation marker, was decreased in kidneys and mRTECs exposed to colistin and this effect was alleviated by 7-HC through the downregulation of HDAC1. In vitro, 7-HC treatment restored histone acetylation at the Nrf2 promoter region and hence promoting Nrf2 expression.

In conclusion, the present study further highlights the protective role of Nrf2 signaling in colistin-induced kidney injury. More importantly, this study is the ﬁrst to reveal that 7-HC could act as HDAC inhibitor and play a crucial role against colistin-induced kidney injury in mice.

## Data Availability Statement

The original contributions presented in the study are included in the article/**Supplementary Material**, further inquiries can be directed to the corresponding author/s.

## Ethics Statement

The animal study was reviewed and approved by Institutional Animal Care and Use Committee of Northeast Agricultural University.

## Author Contributions

JW, CC, and JL designed the study and wrote the paper. JW, MI, and QF finished experiments.

## Funding

This work was supported by the National Natural Science Foundation of China (31472240 and 31802241), Project of Youth Innovative Talent Training Program in Heilongjiang Province (UNPYSCT-2018146), Post-doctoral Research Foundation in Heilongjiang Province (LBH-Z19006) and Academic Backbone Project of Northeast Agricultural University (18XG23).

## Conflict of Interest

The authors declare that the research was conducted in the absence of any commercial or financial relationships that could be construed as a potential conflict of interest.
