# Supplementary material for: 7-Hydroxycoumarin Attenuates Colistin-Induced Kidney Injury in Mice Through the Decreased Level of Histone Deacetylase 1 and the Activation of Nrf2 Signaling Pathway
Source: Front Pharmacol. 2020 Jul 28;11:1146. doi: 10.3389/fphar.2020.01146 (PMC7399215; doi:10.3389/fphar.2020.01146)
Supplement: Supplementary file 1 [file DataSheet_1.docx]

**
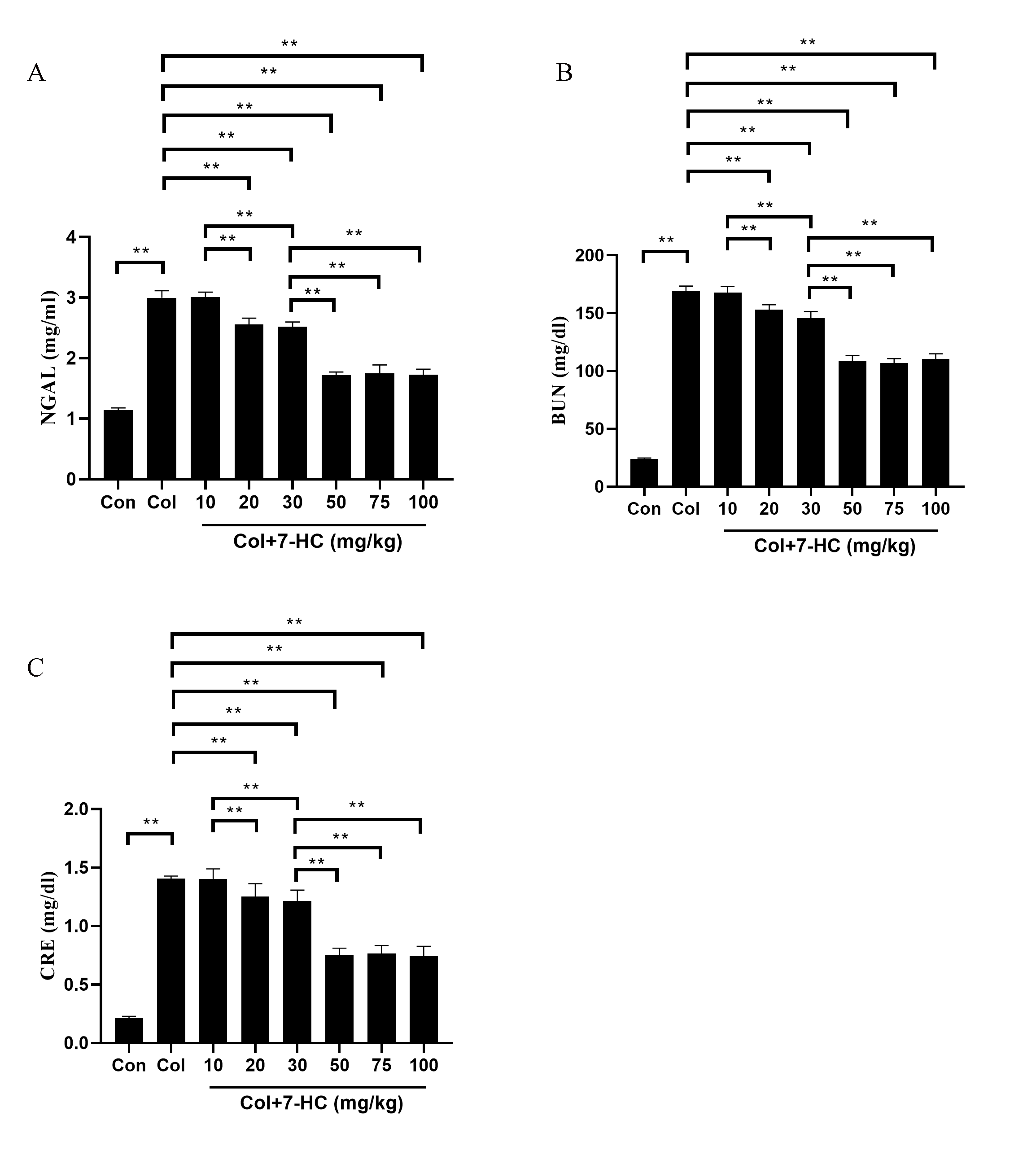
**

**Supplementary Figure 1** Effect of 7-HC on the biomarkers of renal injury induced by colistin. (A-C) Concentrations of serum NGAL, BUN and CRE, respectively (n = 6). Values are the mean ± SD that are significantly different indicated by asterisks as follows: ^**^*P* < 0.01. Con, control; Col, colistin.


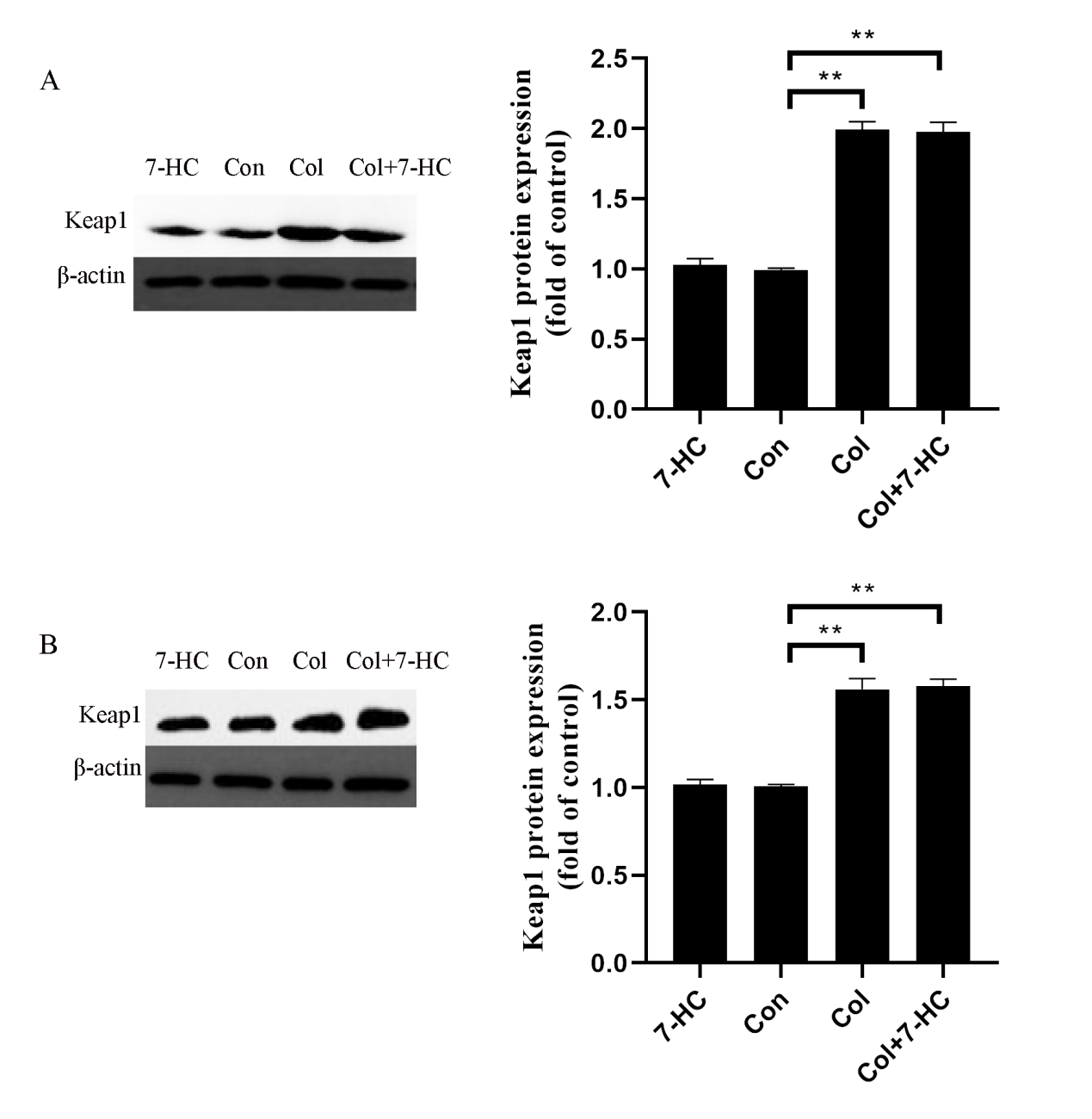


**Supplementary Figure 2** Effect of 7-HC on Keap1 protein expression. (A) Protein expression of Keap1 in the kidney tissues of mice (n = 3). (B) Protein expression of Keap1 in mRTECs (n = 3). Values are the mean ± SD that are significantly different indicated by asterisks as follows: ^**^*P* < 0.01. Con, control; Col, colistin.

.
