# Supplementary figures and images for: 7-Hydroxycoumarin Attenuates Colistin-Induced Kidney Injury in Mice Through the Decreased Level of Histone Deacetylase 1 and the Activation of Nrf2 Signaling Pathway
Source: Front Pharmacol. 2020 Jul 28;11:1146. doi: 10.3389/fphar.2020.01146 (PMC7399215; doi:10.3389/fphar.2020.01146)

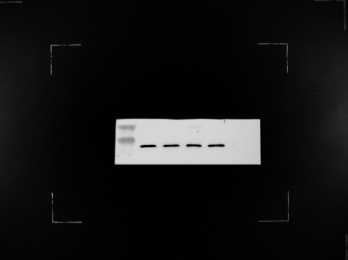

Supplement: Supplementary file 2 [file DataSheet_2.zip › uncropped WB image/FIG 4A actin.tif]

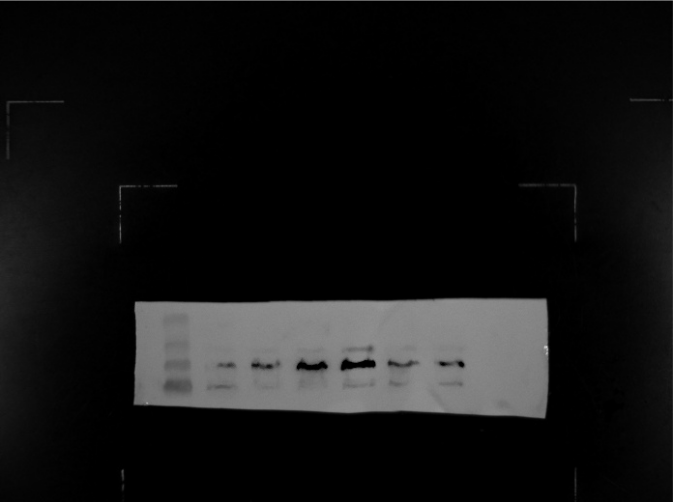

Supplement: Supplementary file 2 [file DataSheet_2.zip › uncropped WB image/FIG 4A HDAC1.tif]

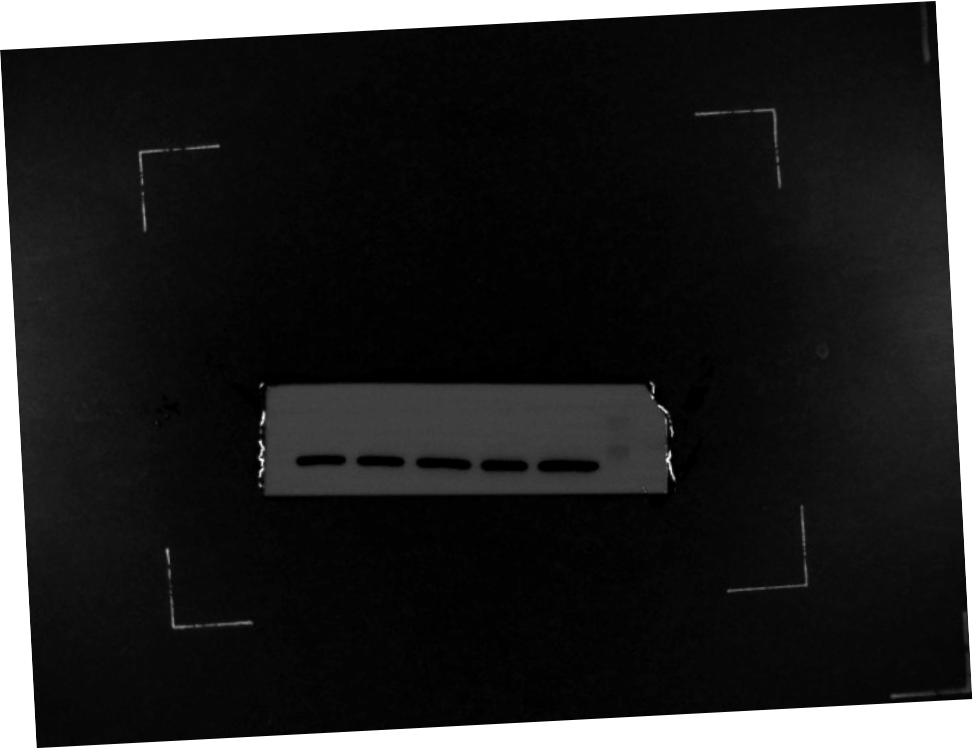

Supplement: Supplementary file 2 [file DataSheet_2.zip › uncropped WB image/FIG 4H actin.tif]

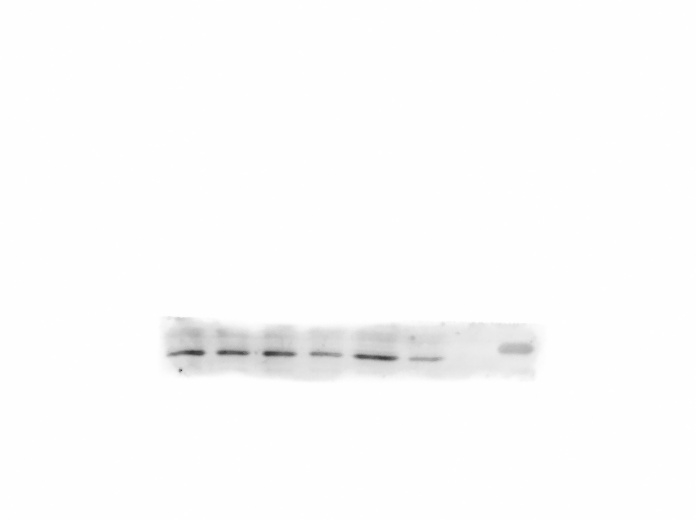

Supplement: Supplementary file 2 [file DataSheet_2.zip › uncropped WB image/FIG 4H H3K27AC.tif]

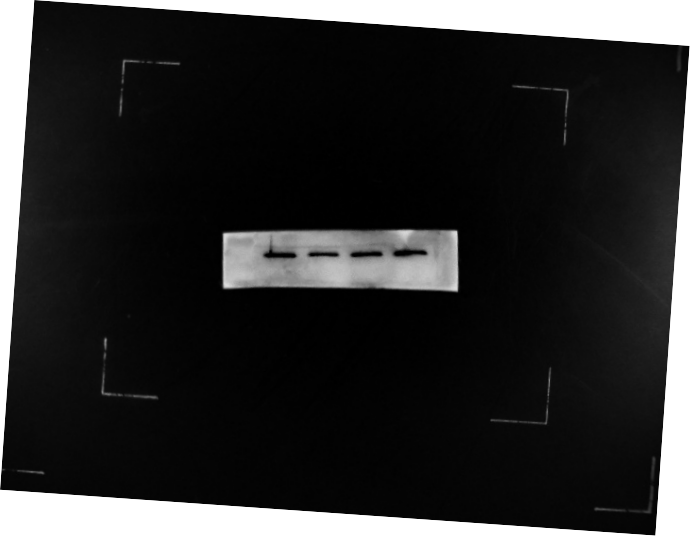

Supplement: Supplementary file 2 [file DataSheet_2.zip › uncropped WB image/FIG 4H Lamin B.tif]

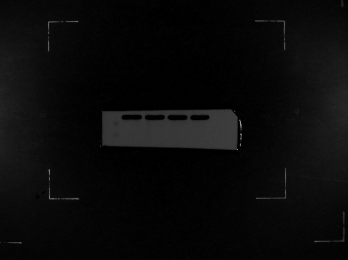

Supplement: Supplementary file 2 [file DataSheet_2.zip › uncropped WB image/FIG 5H actin.tif]

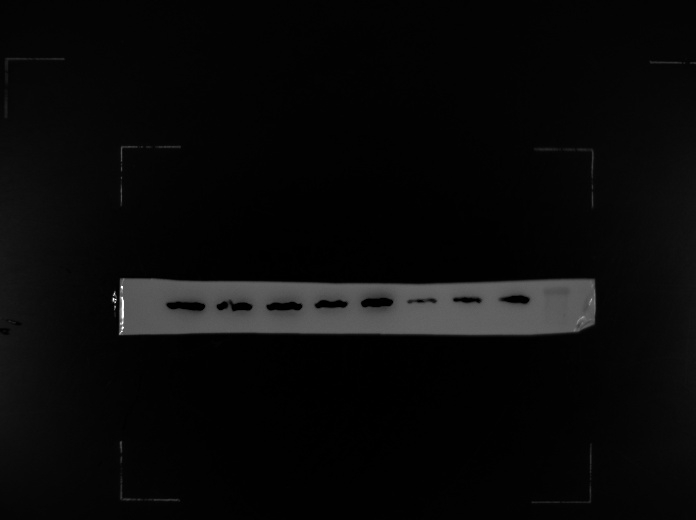

Supplement: Supplementary file 2 [file DataSheet_2.zip › uncropped WB image/FIG 5H HO-1.tif]

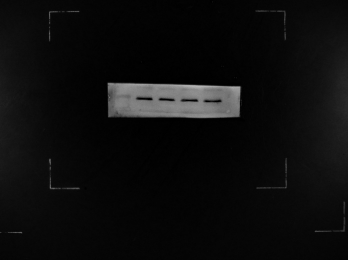

Supplement: Supplementary file 2 [file DataSheet_2.zip › uncropped WB image/FIG 5H Lamin B.tif]

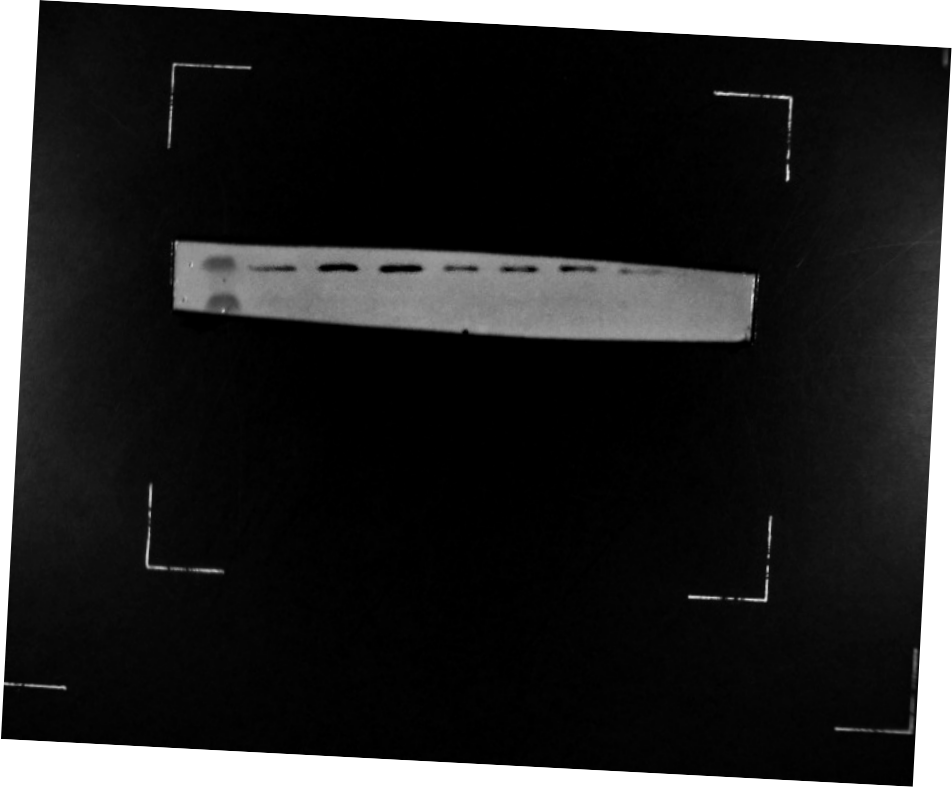

Supplement: Supplementary file 2 [file DataSheet_2.zip › uncropped WB image/FIG 5H Nrf2.tif]

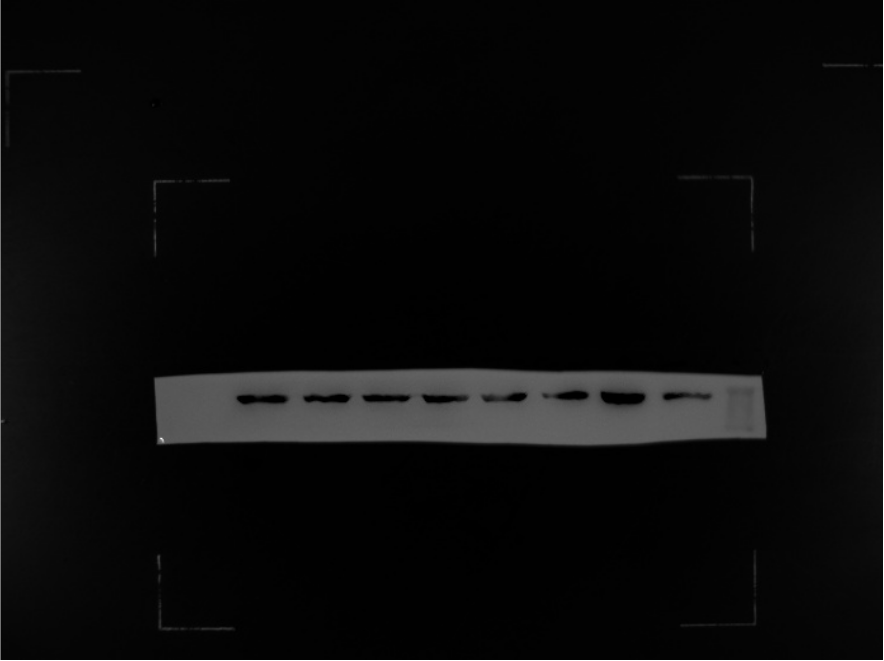

Supplement: Supplementary file 2 [file DataSheet_2.zip › uncropped WB image/FIG. 4H HDAC1.tif]

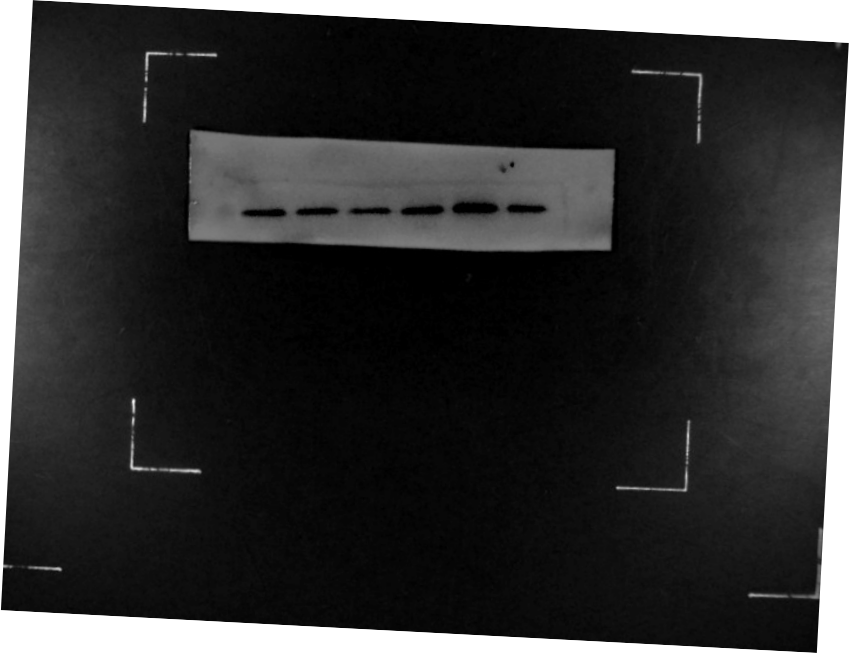

Supplement: Supplementary file 2 [file DataSheet_2.zip › uncropped WB image/FIG. 4H HO-1.tif]

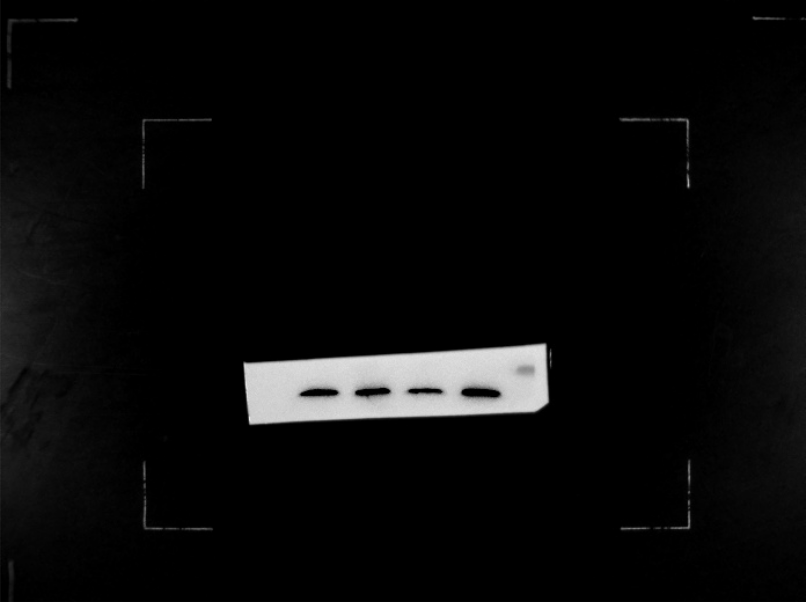

Supplement: Supplementary file 2 [file DataSheet_2.zip › uncropped WB image/FIG. 4H Nrf2.tif]

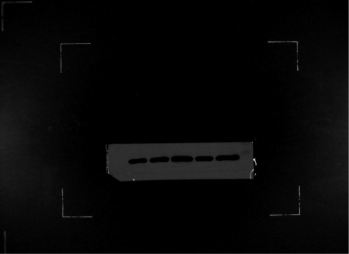

Supplement: Supplementary file 2 [file DataSheet_2.zip › uncropped WB image/FIG.2F actin.tif]

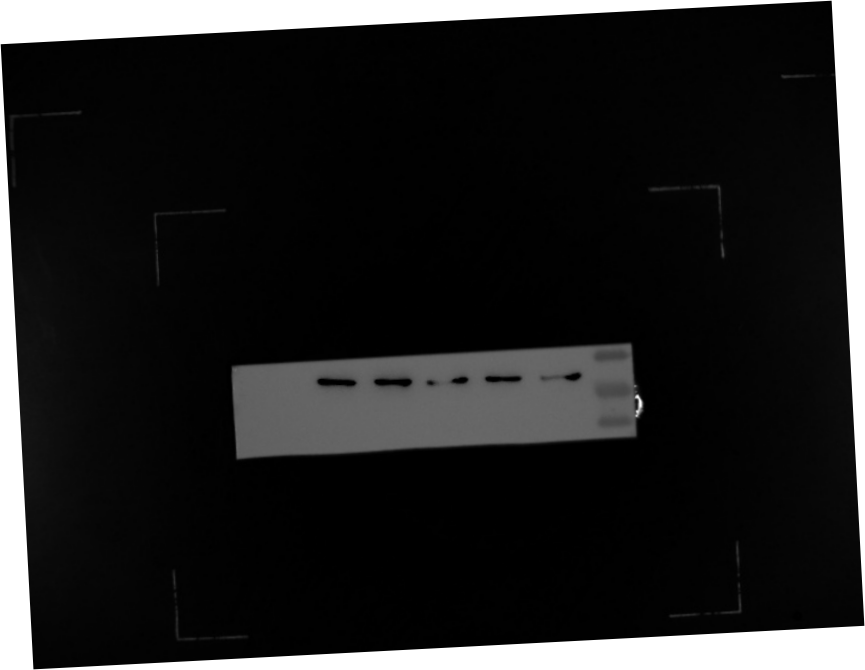

Supplement: Supplementary file 2 [file DataSheet_2.zip › uncropped WB image/FIG.2F HO-1.tif]

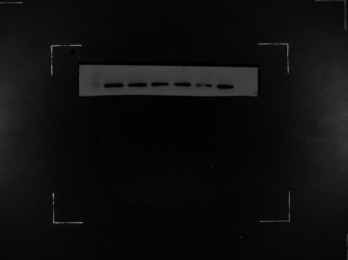

Supplement: Supplementary file 2 [file DataSheet_2.zip › uncropped WB image/FIG.2F Nrf2.tif]

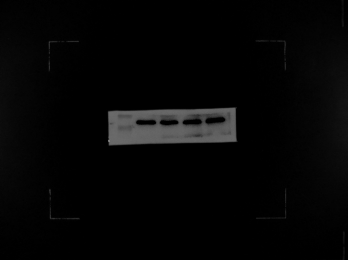

Supplement: Supplementary file 2 [file DataSheet_2.zip › uncropped WB image/FIG.3D actin.tif]

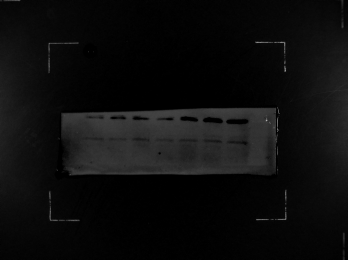

Supplement: Supplementary file 2 [file DataSheet_2.zip › uncropped WB image/FIG.3D H3K27AC.tif]

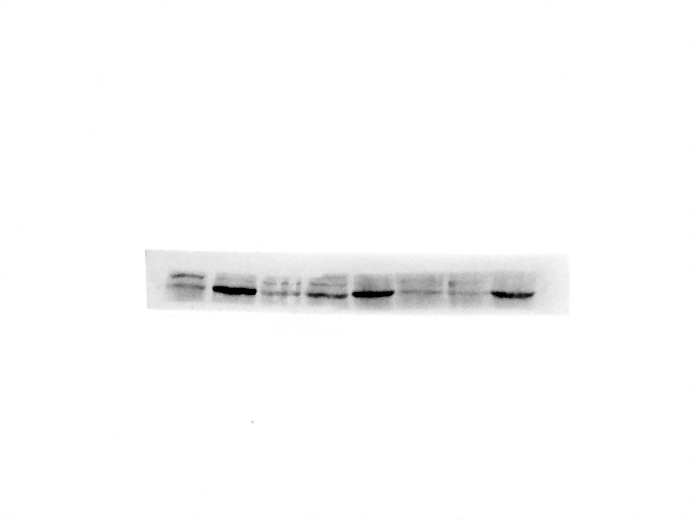

Supplement: Supplementary file 2 [file DataSheet_2.zip › uncropped WB image/FIG.3D HDAC1.tif]

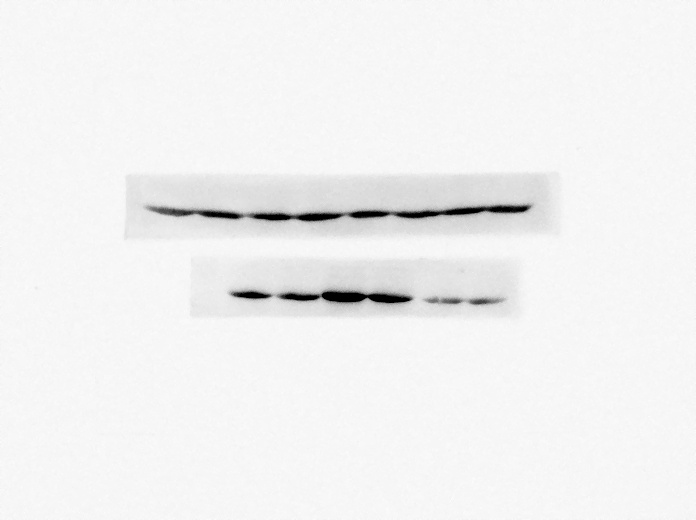

Supplement: Supplementary file 2 [file DataSheet_2.zip › uncropped WB image/Supplementary FIG 2A Keap1.tif]

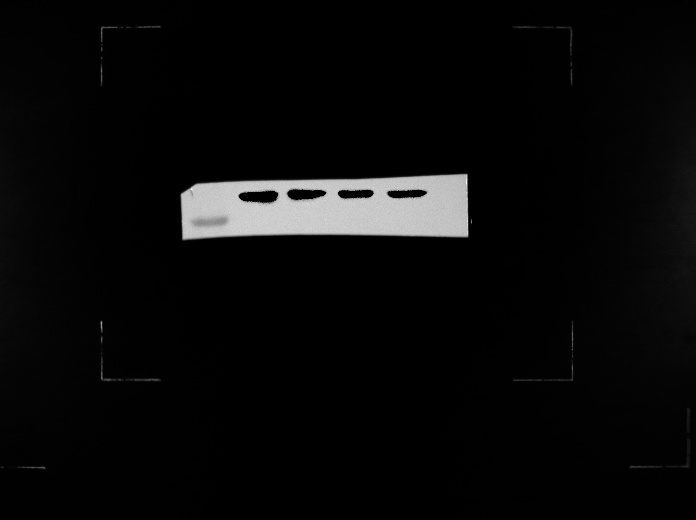

Supplement: Supplementary file 2 [file DataSheet_2.zip › uncropped WB image/Supplementary FIG 2B Keap1.tif]
